# Impact of Infant and Maternal Factors on Energy and Macronutrient Composition of Human Milk

**DOI:** 10.3390/nu12092591

**Published:** 2020-08-26

**Authors:** Agnieszka Bzikowska-Jura, Piotr Sobieraj, Dorota Szostak-Węgierek, Aleksandra Wesołowska

**Affiliations:** 1 Department of Clinical Dietetics, Faculty of Health Sciences, Medical University of Warsaw, E Ciolka Str. 27, 01-445 Warsaw, Poland; dorota.szostak-wegierek@wum.edu.pl; 2 Deparment of Internal Medicine, Hypertension and Vascular Diseases, Faculty of Medicine, Medical University of Warsaw, Banacha Str 1a, 02-097 Warsaw, Poland; piotr.sobieraj@wum.edu.pl; 3 Department of Neonatology, Laboratory of Human Milk and Lactation Research at Regional Human Milk Bank in Holy Family Hospital, Faculty of Health Science, Medical University of Warsaw, Zwirki i Wigury Str. 63A, 02-091 Warsaw, Poland; aleksandra.wesolowska@wum.edu.pl

**Keywords:** human milk composition, breastfeeding, sex-specificity, maternal nutrition, maternal nutritional status, body composition analysis

## Abstract

The present study investigates the influence of selected infant and maternal factors on the energy and macronutrient composition of mature human milk (HM). The study enrolled 77 mothers at 4–8 weeks postpartum. Each mother provided 1 sample of HM. Each extracted HM sample was formed by mixing four subsamples of HM, each of which were obtained in one predefined 6-h periods of the day. Among maternal factors, the analysis included: anthropometric data before and after pregnancy; weight gain in pregnancy; body composition, assessed using the Maltron BioScan 920-II to analyze bioimpedance; and dietary intake, assessed with three-day dietary records. Among the neonatal factors, birth weight and length, number of daily feedings and type of delivery were included. The composition of HM, including energy content, protein, fat and carbohydrate concentrations, was analyzed using the Miris human milk analyzer. Pearson’s and Spearman’s correlation coefficients and multivariable logistic regression models were used to analyze the association between the selected maternal and infant factors and HM milk composition. It was found that total protein content of HM was correlated with pre-pregnancy BMI (Spearman rho = 0.238; *p* = 0.037), current lean body mass (Spearman rho = −0.293, *p* = 0.01) and total water content (Spearman rho = −0.315, *p* = 0.005). Carbohydrates were the only macronutrients whose composition was significantly affected by the infant factors. It was reported that higher carbohydrate content was associated with male sex (OR = 4.52, *p* = 0.049). Our results show that maternal and infant factors, especially maternal pre-pregnancy and current nutritional status and infant sex, interact and affect HM composition, suggesting that macronutrient and energy content in HM may be determined in pregnancy and may have unique compositional profile for every mother–infant dyad.

## 1. Introduction

Human milk (HM), with its unique combination of all essential nutrients and bioactive factors, is a gold standard for pre-term and term infant nutrition. Exclusive breastfeeding for the first six months is recommended by the World Health Organization (WHO) and many national authorities, including that in Poland [1,2,3]. The European Society for Pediatric Gastroenterology Hepatology and Nutrition states that exclusive breastfeeding for around six months is a desirable goal and simultaneously indicates that partial breastfeeding or breastfeeding for shorter periods of time are also valuable [4]. Breast-fed infants have been shown to be better protected than formula-fed infants against serious diseases, including multiple cardiovascular diseases, type 1 and type 2 diabetes mellitus, obesity, necrotizing enterocolitis, inflammatory bowel disease, allergy and asthma [5,6]. Furthermore, results of several studies suggest that breastfeeding has clear short term benefits, particularly reducing morbidity and mortality due to infectious diseases in childhood (e.g., diarrhea and respiratory tract infections). These benefits have been reported in low-, middle- and high-income countries [7].

The HM composition is dynamic and may vary according to a variety of factors related to mother, infant, environment and physiology. The impact of physiological factors, e.g., sequential stages of lactation [8,9], pre-/post-feed [10] and daytime [11] is the best investigated. Changes in maternal diet [12,13,14] and nutritional status [15,16] also significantly influence HM composition, mainly within the lipid fraction. Likewise, the concentrations of non-nutritive milk components (e.g., hormones, steroids, growth and satiety factors) are affected by maternal factors [17]. Curiously, some of the bioactive compounds (e.g., growth hormones and leptin) have been reported to follow a sexually dimorphic pattern of secretion that influences their serum concentration [18,19]. Recent studies show that human and other mammalian milk macronutrient composition may also be tailored for the sex of the offspring. For example, in a bovine model, mothers have been shown to produce a considerably larger volume of milk with higher energy content for their female offspring [20]. On the contrary, Iberian red deer mothers of male calves were observed to produce more milk and of higher protein, fat and lactose content, resulting in higher energy content [21]. In turn, ruminant and marsupial models have demonstrated that mothers of male offspring produce milk higher in protein [20,21,22,23].

Despite data derived from animal models, there remains a dearth of results concerning potential sex specificity in HM composition. Only several human studies have reported an association between HM energy content and macronutrient concentration and infant sex, and due to methodological limitations, their results are incoherent. However, these results are derived from clinical twin research, underpinning a need for sex-specific nutritional strategies for male and female offspring in order to ensure their optimal growth and development. Kanzawa and Segal [24] reported that sex bias suggests that opposite-sex twins, who receive HM that cannot simultaneously be adjusted for both sexes, may be at a disadvantage for growth compared with same-sex twins. Results from the National Longitudinal Study of Adolescent Health [25] show that controlling for sex, age, birth weight, and zygosity, breastfed same-sex twins are, on average, about 12 pounds heavier and 1 inch taller than their opposite-sex counterparts through adolescence and early adulthood. In contrast, never-breastfed same-sex twins tend to be lighter and shorter than their opposite-sex counterparts. These results indicate a supposable impact of infant sex on HM composition and its long-term effects.

The influence of infant sex and maternal factors on HM composition do not have well-defined effects, mainly due to many limitations of previous studies, including no specific day time for milk expression, different lactational stage and using separate analytical instruments for each macronutrient [26,27]. Therefore, we investigated the contribution of infant factors (sex and anthropometric parameters) and maternal factors (e.g., diet and nutritional status) with the energy and macronutrient composition of mature HM, using technologically advance devices for HM and maternal nutritional status analysis.

## 2. Materials and Methods

### 2.1. Participants and Study Design

The present prospective study was based on data and HM samples from 77 exclusively breastfeeding mothers. Participants were enrolled during their 4–8 weeks of lactation from the Department of Obstetrics and Gynecology in Holy Family Hospital and Institute of Mother and Child in Warsaw, Poland. Inclusion criteria involved: age ≥ 18 years, singleton and full-term pregnancy, no gestational diseases (e.g., diabetes mellitus), no smoking during and after pregnancy, sufficient milk supply and infants’ birth weight ≥2500 g. The study session consisted of three parts.

Firstly, we collected data regarding maternal characteristics, such as age, education level, economic status and pre-pregnancy anthropometric parameters (weight and height) and total weight gain during pregnancy. We also asked about newborns’ sex, birth weight and length.

Secondly, we collected information concerning maternal nutrition using three-day dietary records. Sizes of food portions, declared in dietary records, were verified using “Album of Photographs of Food Products and Dishes”, from the National Food and Nutrition Institute in Poland [28]. Energy and nutritional values of maternal diets were calculated using Dieta 6.0 nutritional software (National Food and Nutrition Institute, Warsaw, Poland).

Finally, the actual body weight and height were measured using a Seca 799 measurement station and column scales (±0.1 kg/cm; Seca, Chino, CA, USA). The pre-pregnancy and actual body mass index (BMI) were calculated, and interpretation of the results followed the classification proposed by the WHO [29]. Additionally, in all participants, the analysis of body composition by bioelectrical impedance was performed. All measurements were made with a validated protocol, as discussed in the previous study [12].

This study was approved by the Ethics Committee of The Medical University of Warsaw (KB/172/115) and informed consent was obtained from all women participating in the study.

### 2.2. Milk Sample Collection

For a representative sample for nutritional analysis, 24 h collection sampling was performed. All participants collected samples at home after they were given detailed instructions on taking, storing, and transporting samples to the Regional Human Milk Bank or Nutrition Department of Institute of Mother and Child in Warsaw, Poland. Following the initial milk collection, samples were stored in a refrigerator. Pre- and post-feed samples were collected from all women from four time periods (06:00–12:00, 12:00–18:00, 18:00–24:00, 24:00–06:00) to minimize possible circadian influences on the milk macronutrient composition and energy content. At each time point, 5–10 mL of pre- and post- feed milk samples were obtained from the breast(s) the infant fed from by breast pump or manually. The total volume of sample from every woman during the 24 h period was 20–40 mL. After finishing the 24 h collection period, the samples were stored at −20 °C for further analysis.

### 2.3. Human Milk Composition

The contents of lactose, fat and protein in HM were determined using the MIRIS human milk analyzer (HMA) (Miris, Uppsala, Sweden) with a validated protocol. The accuracy of this device has been validated in previous milk analytical studies [30,31]. The analysis was based on semisolid mid-infrared (MIR) transmission. The HMA provided a calculation of energy using conversion factors of 4.0, 9.25, 4.4 kcal per 100 mL for lactose, fat and protein, respectively. “Total protein” is the protein content based on the total amount of nitrogen in a sample, whereas “true protein” is corrected for non-protein nitrogen compounds, and reflects only the content of actual protein, thus the “true” denotation. The MIRIS HMA uses the factor 6.38 to convert nitrogen content to protein content.

As directed by the manufacturer, the check procedure with zero level adjustment was performed before analysis and after every 10th analysis. Before analysis, all samples were warmed to 40 °C. Human milk is an oil-in-water emulsion, and fat separation (creaming and oiling-off) and protein aggregation are processes common in stored—particularly frozen—milk. This can be due to a slow freezing process or long storage time (age gelation) of milk spectroscopy. For the reduction of such effects, each sample was homogenized for 1.5 s/1 mL of probe using the MIRIS Ultrasonic Processor (milk homogenizer, Miris, Uppsala, Sweden). From each pool, three samples (~10 mL in total) were taken to analyze the nutritional value, and for the final result, we used the average of three measurements.

### 2.4. Statistical Analysis

Continuous variables are presented as means and standard deviation and/or median and interquartile range. The assumption regarding the normality of the analyzed variables was verified using the Shapiro–Wilk test. Discrete variables are presented as number and percentage. The t-Student or Mann–Whitney test was used for comparisons of continuous variables between the groups, depending on the distribution. The χ^2^ test was employed for comparison of discrete variables between the groups. The differences were considered as significant when the *p*-value was lower than 0.05.

To establish the association between the analyzed variables and HM composition, Spearman and Pearson correlations were used. When both correlated variables had normal distribution (on the basis of the Shapiro–Wilk test), Pearson’s correlation coefficient was calculated, otherwise Spearman’s coefficient was used. Multivariable logistic regression models were built to assess the influence between multiple maternal factors (current BMI, body composition and macronutrients contribution in the diet), infant factors (sex and birth weight, number of feedings daily), type of delivery and composition of HM. The dependent variable in the models was coded as dichotomous using the value of the 5th quintile of each component of HM as a threshold. The least absolute shrinkage and selection operator (LASSO) method was used for variable selection and regularization of the models. Results of multivariable logistic regression were presented as odds ratio (OR) with a 95% confidence interval (CI) for 1-unit increments of each variable (except birth weight—100 g was selected as the increment interval). All computations were performed in R 3.6.0 (R Foundation for Statistical Computing, Vienna, Austria), an environment for statistical programming.

## 3. Results

### 3.1. Study Population

We collected data from 77 mother–infant pairs. The characteristic of the study participants and HM composition is presented in Table 1. All participants declared a high university education and high (*n* = 40; 52%) or middle (*n* = 37; 48%) socioeconomic status. The mean maternal age was 32.4 ± 4.4 years, and most participants were primiparous (*n* = 47; 61%). Before pregnancy and during the first weeks of lactation, most of the women had normal mass (*n* = 56; 73%; *n* = 55; 71%, respectively). In 19% cases pre-pregnancy, the BMI value was classified as overweight or obese (*n* = 15). Among the 77 mothers who participated in the study, 42 delivered a son (55%) and 35 delivered a daughter (45%).

### 3.2. Comparison of Subjects According to Newborn’s Sex

Table 2 presents detailed comparison of mothers according to newborn’s sex. The majority of mothers who delivered a boy declared cesarean section (*n* = 29, 64%), whereas this was require for only 29% mothers of girls (*p* = 0.004). Compared to mothers of boys, mothers with female infants had a higher pre-pregnancy weight (*p* = 0.022), current weight (*p* = 0.030), pre-pregnancy BMI (*p* = 0.004) and current BMI (*p* = 0.042). However, differences in body composition between the groups were not found. Analyzing nutritional value of daily food rations, mothers with male infants tended to consume a higher amount of vegetable protein (*p* = 0.045) and fiber (*p* = 0.016) compared to those with female infants.

### 3.3. Infants’ Characteristics and Human Milk Composition

Table 3 presents infants’ characteristics and HM composition according to infants’ sex. As shown, we did not observe any statistically significant differences in anthropometric data and the number of daily feedings among male and female infants. In the case of HM composition, the liquid energy density of milk produced by mothers of female infants was 63.9 kcal/100 mL versus 63.7 kcal/100 mL of milk produced by mothers of male infants (*p* > 0.05). However, we reported that mothers of male infants produce milk with a higher carbohydrate content (*p* = 0.027).

### 3.4. Maternal and Infant Factors and Human Milk Composition

Table 4 presents correlations between maternal factors, maternal diet, infant factors and HM composition. We found that carbohydrate HM content was positively correlated with the number of daily feedings (Spearman rho = 0.308, *p* = 0.007).

Total protein content of HM was correlated with pre-pregnancy BMI (Spearman rho = 0.234; *p* = 0.037) and current lean body mass (Pearson rho = −0.293, *p* = 0.01). However, when true protein content was analyzed, it was correlated with current lean body mass (Pearson rho = −0.267, *p* = 0.019), total water content (Spearman rho = −0.292, *p* = 0.01) and vegetable protein intake (Spearman rho = 0.23, *p* = 0.045).

Pre-pregnancy BMI (Spearman rho = 0.276, *p* = 0.006), current BMI (Spearman rho = 0.351, *p* = 0.002), lean body mass (Pearson r = −0.330, *p* = 0.002) and total water content (Spearman rho = −0.323, *p* = 0.004) were correlated with fat content in HM. 

We found correlations between pre-pregnancy BMI, current BMI, lean body mass, total water content and HM energy content (Spearman rho and *p*-values: 0.296, 0.001; 0.378, 0.001; −0.355, 0.002; −0.329, 0.003, respectively).

To evaluate the relationship between the analyzed maternal and infant factors, multivariable logistic regression models were built, but firstly the 5th quintiles of carbohydrates, total protein, true protein, fat and energy in HM were calculated (7.27 g/100 mL, 1.23 g/100 mL, 1.00 g/100 mL, 4.4 g/100 mL, 73.9 kcal/100 mL, respectively).

The relationship between infant’s sex and HM components was evaluated in a multivariable logistic regression model including birth weight, number of daily feedings, and fat percentage component of daily energy delivery (Table 5, Model A). Male infant sex was associated with high carbohydrate HM content, (OR = 4.52, 95% CI 1.09–23.56). Higher current birth weight, the number of daily feedings and the fat percentage component of daily energy delivery were associated with higher OR for high carbohydrate content of HM.

We did not find a relationship between infants’ sex and high concentration of protein (Model B and C) or fat (Model D). However, according to Model B, higher concentration of total protein in HM may be expected in older mothers who had lower weight gain during pregnancy and higher postpartum weight. Higher true protein concentration in HM was associated with higher age and higher current BMI. Higher current BMI, higher body fat tissue, higher protein and carbohydrate percentage component of daily energy delivery were associated with HM rich in fat (Model D). None of the analyzed newborn factors influenced the protein or fat content in HM.

Despite this, in the multivariable logistic regression model, we observed the tendency that male newborns had higher probability to receive HM rich in energy (OR 5.23, 95% CI 0.98–37.8), thus, the impact of newborns’ sex had marginal statistical significance (*p* = 0.070, Model E). A similar tendency was observed when the association between the number of daily feedings and energy content of HM was analyzed (*p* = 0.099). Other factors associated with maternal status also influenced the energy content of HM. Higher current BMI, body fat tissue, the number of daily feedings and the protein percentage component of daily energy delivery were associated with HM rich in energy (Model E). Figures S1–S5 picture plots presenting correlation between carbohydrates (S1), total protein (S2), true protein (S3), fat (S4), energy content (S5) in HM and selected maternal and infant factors.

## 4. Discussion

In this population, we observed an association between carbohydrate concentration in HM and infant sex. We reported that mothers of male infants produce HM with greater carbohydrate content. Simultaneously, we did not find a relationship between infants’ sex and other macronutrients in HM. What is more, our findings showed an association between current BMI, maternal body composition during breastfeeding and infant sex and the macronutrient concentration in mature HM, collected 1–2 months postpartum. Similar to other studies [16], our data show that fat concentration in HM was positively correlated with maternal nutritional status. However, our study provides better insight by assessing nutritional status not only with BMI value, but mainly with body composition analysis and analyzing milk samples from a 24-h period.

Fat concentration of HM was described across the studies as the most variable component. The majority of studies that examined associations found a positive correlation between maternal BMI and/or body fat tissue and HM fat concentration, providing further support of higher levels of fat in HM in mothers with higher BMI [32]. The roles of external factors in regulating fat synthesis in the breasts are still not well understood [27]. It is suggested that metabolic disorders (e.g., dyslipidemia, hypertriglyceridemia, type 2 diabetes), commonly reported in women with a higher fat mass, are associated with an increase in fat concentration in HM [33]. An alternative possibility is that the higher fat concentration in HM results from higher dietary fat intakes in women who are overweight or obese. However, the majority of researchers did not find evidence for associations between the maternal intake of any dietary nutrients and the milk composition, including fat concentration [12,34]. In our study, we found that pre-pregnancy BMI was positively correlated with fat concentration in HM (*p* = 0.006), whereas lean body mass (*p* = 0.002) and total water content (*p* = 0.004) were correlated negatively. A decreased body water is common in overweight and obese women, explaining this negative correlation. Findings of previous studies [35,36] based on maternal BMI are consistent with our results. Chang et al. [35] observed that women’s current BMI was positively correlated with fat concentration in HM at early (1–2 weeks) and late (7–8 months) lactation stages. However, Leghi et al. [16] reported in their meta-analysis that the fat concentration in transitional milk showed an inverse correlation. Simultaneously, no impact of maternal overweight or obesity on fat concentration in colostrum was observed. The fact that maternal nutritional status had less impact on HM fat fraction at earlier stages of lactation is consistent with the results of the DARLING study (Davis Area Research on Lactation, Infant Nutrition and Growth) [27], which suggested that HM composition is more sensitive to maternal factors (including nutritional status) in later lactation that during the first few weeks postpartum. The first days and weeks postpartum are connected with rapid mobilization of pregnancy fat tissue stores and dynamic shifts in HM composition. Thus, it may be difficult to identify the influence of other external factors affecting HM composition until later on in lactation [27]. We did not find any correlations between infant factors and fat content in HM milk, whereas Hahn et al. [37] reported that infant length was negatively associated (OR = 0.84, *p* = 0.004). Interestingly, Fujita et al. [38] reported association between female sex and higher fat content in HM in mothers with low socioeconomic status, confirming the Trivers–Willard hypothesis which predicts the unequal parental investment between daughters and sons, depending on maternal condition and offspring reproductive potential. Furthermore, Fischer Fumeaux et al. [39] found “sex biases” in HM composition. They indicated that male gender of infants was significantly and independently associated with higher energy content and fat concentration in HM. This observation was made in term and pre-term infants, but it was more marked in the pre-term group. These results are consistent with study conducted in Singapore. Thakkar et al. [40] reported that HM for male infants compared to females at 120 days postpartum was higher in energy and fat by 24% and 39%, respectively.

The impact of maternal and infant factors on HM protein concentration remains inconclusive. In our study, we observed that total protein content of HM was significantly positively correlated with pre-pregnancy BMI (*p* = 0.037) and negatively correlated with current lean body mass (*p* = 0.01) and total water content (*p* = 0.005). In the multivariable logistic regression model, maternal current BMI was found to be associated with HM total and true protein content (OR = 1.36, *p* = 0.003; OR = 1.23, *p* = 0.022, respectively). In previous studies, it was reported that maternal obesity is associated with impairments in amino acid metabolism, resulting in raised amino acid concentrations in HM [41] and circulation [42,43,44]. Furthermore, it was also observed that HM protein concentration in mature milk was positively correlated with infant birth weight [42] and infant BMI at 1 year postpartum [45]. An Australian study [46] including 20 breastfeeding dyads showed that casein concentration in HM and calculated daily intakes of casein in infants were positively associated with lower infant fat-free mass (*p* = 0.003, *n* = 18), higher fat mass (*p* < 0.001), fat mass index (*p* = 0.001, *n* = 18), and % fat mass (*p* < 0.001, *n* = 18) measured with ultrasound skinfolds. Grunewald et al. [47] confirmed these results. They studied 196 pairs of mothers and infants from a European research project (PreventCD) and found that one-month milk protein content was strongly associated with infant serum lyso-phosphatidylcholine (LPC) 14:0 (linear mixed effect model corrected for multiple testing—_P_LME = 0.009). Thus, infant serum LPC was influenced by breast milk fatty acids composition and, intriguingly, milk protein content in early but not late lactation. Interestingly, the authors also indicated that LPC 14:0 was previously positively associated with obesity risk in children. All these results suggest that protein concentration in HM plays a significant role in the programming of metabolism and growth in the future life. Similarly to other studies [37,48], we did not observe any association between HM protein content and infants’ sex and birth anthropometric measurements.

The analysis of carbohydrates by MIRIS HMA is affected by the presence of lactose and nonlactose carbohydrates, mainly human milk oligosaccharides (HMOs) [30]. Some of the divergence between the findings for lactose concentrations may be related to the inclusion of HMOs in the mid-infrared (mid-IR) transmission spectroscopy measurements [31]. Since the reference laboratory analysis for lactose concentration, high-pressure liquid chromatography (HPLC), does not measure HMOs, it is probable that lactose levels measured by mid-infrared transmission spectroscopy were a result of absorbing terminal or core lactose moieties of HMOs [49]. The measured concentration of carbohydrates in our study (6.9 g/100 mL) was consistent with the normal range in human milk [9]. In our study, carbohydrates were the only macronutrients whose composition was significantly affected by infant factors. We found that higher carbohydrate content was associated with male sex (OR = 4.52, *p* = 0.049) and the number of daily feedings (OR = 1.27, *p* = 0.013), whereas birth weight was associated with lower carbohydrate content in HM (OR = 0.89, *p* = 0.147). In contrast, Hahn et al. [37] reported higher carbohydrate content for females (OR = 0.56, *p* = 0.012). They also found that a lower concentration of carbohydrates in HM was related to cesarean section (OR = 0.65, *p* = 0.046). This result indicates that the hormonal activity induced by labor and uterine contraction may account for the alteration in HM carbohydrate content. These findings may support the association between infant gender and carbohydrate content in HM. To the best of our knowledge, only our and Hahn et al.’s [37] study reported this association; therefore, these unique and unclear results should be further investigated. Our result, concerning positive correlation between the number of daily feedings and HM lactose concentration, is consistent with that of Dewey et al. [50] who observed that a decrease in milk volume below 300 mL/day is associated with a lower lactose concentration in HM. The results of the DARLING study are similar [27]. The authors reported a positive correlation between milk volume and lactose concentration at 6 and 9 months postpartum. According to Neville et al. [51] the positive association of milk volume and lactose concentration may reflect the fact that this macronutrient is the major determinant of HM osmolarity and, thus, its synthesis influences the volume of fluid secreted by the mammary gland. They also indicated that the concentration of lactose may be altered by secretion rates of electrolytes that also contribute to osmolarity.

Increased demand for milk from male infants could feasibly result in an increase in the energy density of their mothers’ HM. It has previously been suggested that male infants may consume 8–10% more milk than females [52]. Unfortunately, despite that in our study male sex was associated with a higher probability of receiving high-in-energy human milk (OR 5.23, 95% CI 0.98–37.8), this factor did not reach statistical significance in the multivariable model (*p* = 0.070). Similarly, the number of daily feedings did not reach statistical significance (*p* = 0.099) Furthermore, Powe et al. [53] reported that mothers of male infants produced 25% greater energy content in HM than mothers of female infants. Interestingly, a study of 244 Boston women [54] found that pregnant women carrying male infants consume about 10% more kcal than pregnant women carrying female infants, raising the additional possibility that male offspring place greater demands on their mothers even in utero. In contrast, in our population, mothers of females ate more kcal (mean 1747 ± 494.5 kcal) than mothers of males (mean 1713 ± 412 kcal), although this result did not reach statistical significance (*p* = 0.536). 

The present study provides a comprehensive assessment of the energy and macronutrient composition of HM. To our knowledge, this is one of the few studies that focused on the relationship between a significant number of maternal and infant factors (mainly infants’ sex) and energy content and macronutrient concentration in HM. The procedure of milk sample collection was cautiously planned and performed to minimize errors resulting from physiological factors and subsequently affecting HM composition. The additional strengths of this study are the use of advanced techniques to assess maternal body composition in accordance with the recommended protocol [55], which allowed possible errors in body composition to be minimized. The present study has also some limitations. Firstly, the study was conducted with convenience sampling and a modest number of participants. This limitation is especially troublesome when the influence of newborns’ sex on HM energy content is analyzed. Despite the high odds ratio, the reported factor did not reach statistical significance. We speculate that if the study population was larger, we would have been able to conclude that gender affects the energy content of milk. Secondly, observations made in this investigation are specific to Caucasian populations and should not be generalized to other ethnic groups. Finally, regarding the maternal diet, we did not collect information about nutritional habits during pregnancy and did not standardize the procedure of HM extraction, which may affect HM composition [56,57]. The results of our study are also limited by the fact that in comparison of male and female newborns, differences in maternal factors (weight, body mass index and vegetable protein intake) and newborns (type of delivery) were present. However, multivariable logistic regression models were developed to reduce risk of potential bias.

## 5. Conclusions

Overall, our findings show that maternal and infant factors, especially maternal pre-pregnancy and current nutritional status and infant sex, interact and affect HM composition, suggesting that macronutrient and energy content in HM may be determined in pregnancy and may have unique compositional profile for every mother–infant dyad. Maternal factors affecting HM composition are better investigated, however, there are still only a few studies evaluating the impact of infant birth factors on HM nutritional profile. In our study, male gender appears to be associated with a higher concentration of carbohydrates in HM. In the future, studies assessing HM composition require validation and specification in additional human cohorts. The main objective of these studies should be evaluating variations of HM composition, which is crucial to consider for the optimization of nutrition and growth of infants.

## Figures and Tables

**Table 1:** General characteristics of the study group (*n* = 77).

Parameter	Mean ± Standard Deviation	Median (Lower Quartile–Upper Quartile)	Number (Percentage)
**Mothers**			
Age (years)	32.4 ± 4.4	32 (29–34)	
Pre-pregnancy weight (kg)	61.3 ± 10.7	58.0 (53.5–70.0)	
Pre-pregnancy BMI (kg/m^2^)	22.0 ± 3.5	21.1 (19.3–23.8)	
Pre-pregnancy nutritional status:			
Underweight			6 (8%)
Normal weight			56 (73%)
Overweight			12 (15%)
Obese			3 (4%)
Pregnancy weight gain (kg)	15.0 ± 5.1	14 (12–17)	
Current weight (kg)	64.1 ± 12	62.0 (54.7–70.1)	
Current BMI (kg/m^2^)	23 ± 3.8	22.5 (20–24.9)	
Current nutritional status according to BMI:			
Underweight			5 (7%)
Normal weight			55 (71%)
Overweight			13 (17%)
Obese			4 (5%)
Body fat tissue (%)	37.2 ± 19.7	30.4 (23.8–41.7)	
Lean body mass (%)	71.9 ± 8.2	71.3 (66.9–78.8)	
Total water content (%)	50.9 ± 4.8	50.3 (46.6–54.8)	
Number of pregnancies:			
First			47 (61%)
Second			22 (29%)
Third			5 (6%)
Fourth			3 (4%)
**Infants**			
Birth weight (g)	3608 ± 462.0	3560 (3560–3900)	
Birth length (cm)	55.2 ± 2.1	55 (54–56)	
Number of daily feedings	9.7 ± 3.4	9 (8–11)	
**Milk composition**			
Energy (kcal/100 mL)	63.8 ± 12.2	63.7 (52–73)	
Total protein (g/100 mL)	1.12± 0.22	1.1 (1–1.2)	
True protein (g/100 mL)	0.88 ± 0.18	0.83 (0.8–1)	
Fat (g/100 mL)	3.34 ± 1.23	3.33 (2.5–4.3)	
Carbohydrates (g/100 mL)	6.99 ± 0.37	7 (6.87–7.23)	
Dry mass (g/100 mL)	11.5 ± 1.5	11.6 (10.3–12.6)	
Water (mL/100 mL)	88.5 ± 1.5	88.4 (87.4–89.7)	

BMI—body mass index.

**Table 2:** Comparison of mothers according to newborn’s sex.

	Female Newborns (*n* = 42)		Male Newborns (*n* = 35)		
	Mean ± Standard Deviation	Median (Lower Quartile–Upper Quartile)	Mean ± Standard Deviation	Median (Lower Quartile–Upper Quartile)	*p*-Value ^1^
Age (years)	32.7 ± 4.2	32 (30–34)	32.1–4.6	31 (29–34.5)	0.405
**Maternal nutritional status and body composition**					
Pre-pregnancy weight (kg)	64.6 ± 12	65.0 (55–74.5)	57.4–7.4	57.0 (53–58)	0.022
Pre-pregnancy BMI (kg/m^2)^	23.1 ± 3.9	23.0 (19.8–25.7)	20.8 ± 2.4	19.8 (19.2–21.6)	0.004
Pregnancy weight gain (kg)	15.8 ± 5.2	14.5 (12–19.8)	13.9–4.7	14 (10–15)	0.099
Current weight (kg)	67.1 ± 13.2	67.8 (55.6–79.2)	60.5 ± 9.3	59.0 (54.7–66)	0.030
Current BMI (kg/m^2^)	23.9 ± 4.2	23.3 (20.1–26.8)	21.9 ± 2.9	21.5 (20–23.6)	0.042
Body fat tissue (%)	38.2 ± 21.1	30.7 (25.2–41.7)	36 ± 18.1	30.4 (22.5–38.8)	0.701
Lean body mass (%)	71.2 ± 8.1	69.9 (66.8–77.1)	72.9 ± 8.2	73.3 (67.7–79.6)	0.369
Total water content (%)	50.8 ± 5	50.0 (46.9–54.6)	51.1 ± 4.6	51.3 (46.6–55)	0.642
**Maternal diet**					
Energy (kcal)	1747 ± 412	1724 (1484–1989)	1827 ± 494.5	1713 (1454–2185)	0.536
Total protein (g)	74.2 ± 21.9	71.4 (56.2–91.1)	75.2 ± 19.4	74.4 (59.7–91.1)	0.831
Protein (% kcal)	17.1 ± 3.4	17 (14.3–19.9)	16.8 ± 2.7	16.9 (15.1–18.7)	0.822
Animal protein (g)	47.9 ± 20.2	42.5 (32.8–63.1)	42.9 ± 19.5	42.5 (33.4–56.7)	0.509
Vegetable protein (g)	25.8 ± 8.8	24.6 (21.3–30.1)	31.5 ± 12.4	26.9 (22.8–36.4)	0.045
Fat (g)	61.2 ± 19.3	58.0 (50.2–66.4)	64.6 ± 24.2	60.9 (48.1–78)	0.602
Fat (% kcal)	31.2 ± 5.9	30.3 (27.8–35.2)	30.9 ± 6.1	30.1 (26.6–34.8)	0.842
Carbohydrates (g)	242.2 ± 61	240.9 (193.1–291.5)	258.6 ± 69	249 (211.1–295.5)	0.278
Carbohydrates (% kcal)	51.7 ± 6.5	51.6 (47.9–55.7)	52.3 ± 7.2	51.1 (47.9–57)	0.638
Sucrose (g)	52.5 ± 30.2	45.6 (33.6–60.2)	44.6 ± 31	34.4 (24.1–51.8)	0.072
Fiber (g)	19.2 ± 6	18.2 (16.0–22.5)	24.4 ± 9	22.0 (18.8–29.3)	0.016

BMI—body mass index. ^1^ t-Student or Mann–Whitney tests were used depending on variable distribution.

**Table 3:** The comparison of anthropometric data, feeding frequency and human milk composition in female and male newborns.

	Female Newborns (*n* = 42)		Male Newborns (*n* = 35)		
	Mean ± SD	Median (Lower Quartile–Upper Quartile)	Mean ± SD	Median (Lower Quartile–Upper Quartile)	*p*-Value ^1^
**Infants’ characteristic**					
Birth weight (g)	3661 ± 486	3625 (3268–4035)	3545 ± 431	3500 (3220–3880)	0.273
Birth length (cm)	55.2 ± 2.1	55 (54–56)	55.1 ± 2.1	55 (54–56)	0.938
Number of daily feedings	9.2 ± 3.1	9.0 (8.0–10.0)	10.3 ± 3.8	9.0 (8.0–11.5)	0.200
**Milk composition**					
Energy (kcal/100 mL)	63.9 ± 11.6	65.5 (52–72.8)	63.7 ± 13.1	63.0 (54.0–71.7)	0.921
Total protein (g/100 mL)	1.11 ± 0.22	1.10 (1.00–1.17)	1.13 ± 0.22	1.10 (1.00–1.20)	0.741
True protein (g/100 mL)	0.88 ± 0.18	0.82 (0.75–0.97)	0.88 ± 0.18	0.9 (0.80–1.00)	0.784
Fat (g/100 mL)	3.40 ± 1.16	3.45 (2.50–4.27)	3.27 ± 1.32	3.20 (2.25–4.08)	0.652
Carbohydrates (g/100 mL)	6.90 ± 0.41	6.95 (6.73–7.13)	7.09 ± 0.27	7.13 (6.90–7.30)	0.027 *
Dry mass (g/100 mL)	11.62 ± 1.46	11.90 (10.37–12.66)	11.44 ± 1.55	11.46 (10.25–12.35)	0.600
Water (mL/100 mL)	88.38 ± 1.46	88.10 (87.34–89.63)	88.56 ± 1.55	88.54 (87.65–89.75)	0.600

^1^ t-Student or Mann–Whitney tests were used depending on variable distribution. * *p* < 0.05.

**Table 4:** Correlations between maternal factors, maternal diet, infant factors and HM composition.

	Correlation Coefficient				
Parameter	HM Carbohydrates	HM Total Protein	HM True Protein	HM Fat	HM Energy
**Maternal factors**					
Age (years) ^1^	0.817	0.182	0.123	−0.024	0.024
Pre–pregnancy BMI (kg/m2) ^1^	0.196	0.234 *	0.23 *	0.276 *	0.296 *
Pregnancy weight gain (kg) ^1^	0.3	0.009	−0.096	0.065	0.058
Current BMI (kg/m^2^) ^1^	0.233 *	0.296 *	0.24 *	0.351 *	0.378 *
Body fat tissue (%) ^1^	0.025	0.056	−0.033	0.061	0.012
Lean body mass (%) ^2^	−0.127	−0.293 *	−0.267 *	−0.330 *	−0.355 *
Total water content (%) ^1^	−0.167	−0.315 *	−0.292 *	−0.323 *	−0.329 *
**Maternal diet**					
Energy ^1^	−0.104	−0.024	0.105	0.088	0.077
Total protein (g) ^1^	−0.105	−0.081	0.026	0.14	0.135
Animal protein (g) ^1^	−0.08	−0.12	−0.079 *	0.118	0.11
Vegetable protein (g) ^2^	−0.111	0.047	0.23	0.084	0.052
Fat (g) ^1^	0.089	−0.02	0.102	0.174	0.181
Carbohydrates (g) ^2^	−0.222	−0.01	0.101	0.015	−0.054
Protein (% kcal) ^2^	−0.016	−0.07	−0.098	0.116	0.115
Fat (% kcal) ^2^	0.194	−0.005	0.035	0.114	0.122
Carbohydrates (% kcal) ^2^	−0.157	0.054	0.014	−0.153	−0.156
**Infant factors**					
Birth weight (g) ^2^	−0.074	0.094	0.055	0.098	0.087
Birth length (cm) ^1^	−0.113	0.137	0.19	0.104	0.093
Number of daily feedings ^1^	0.308^*^	0.105	0.145	0.094	0.121

^1^ Pearson’s correlation coefficient. ^2^ Spearman’s correlation coefficient. * *p* < 0.05. HM—human milk, BMI—body mass index.

**Table 5:** Multiple logistic regression models explaining the relationship between maternal and infant factors and HM composition.

Parameter	OR	95% Confidence Interval	*p*-Value
**Model A—M Carbohydrate content > 7.27 g/100 mL**			
Birth weight (100 g)	0.89	0.75–1.04	0.147
Number of daily feedings	1.27	1.07–1.56	0.013
Maternal fat intake (% kcal)	1.16	1.03–1.34	0.023
Male infant sex	4.52	1.09–23.56	0.049
**Model B—HM Total protein content > 1.23 g/100 mL**			
Current BMI^2^ (kg/m^2^)	1.36	1.13–1.71	0.003
Mother’s age (years)	1.13	0.98–1.3	0.077
Pregnancy weight gain (kg)	0.82	0.65–0.99	0.073
**Model C—HM True protein content > 1.0 g/100 mL**			
Current BMI (kg/m^2^)	1.23	1.04–1.48	0.022
Mother’s age (years)	1.13	0.97–1.31	0.098
**Model D—HM Fat content > 4.4 g/100 mL**			
Current BMI^2^ (kg/m^2^)	1.41	1.17–1.77	<0.001
Body fat tissue (%)	1.04	1.01–1.08	0.014
Maternal protein intake (% kcal)	1.15	0.91–1.5	0.256
Maternal carbohydrate intake (% kcal)	0.93	0.83–1.04	0.201
**Model E—HM Energy content > 73.9 kcal/100 mL**			
Current BMI ^2^ (kg/m^2^)	1.50	1.21–1.99	0.001
Body fat tissue (%)	1.04	1.00–1.08	0.033
Number of daily feedings	1.17	0.98–1.44	0.099
Maternal protein intake (% kcal)	1.32	1.05–1.74	0.027
Male infant sex	5.23	0.98–37.8	0.070

The results are presented using odds ratio with 95% confidence intervals. Odds ratios were computed for 1-unit increases in each variable except birth weight (increment interval 100 g). HM—human milk, BMI—body mass index, OR—odds ratio.

## Supplementary Materials

The following are available online at https://www.mdpi.com/2072-6643/12/9/2591/s1, Figure S1. Plots presenting correlation between carbohydrates concentration in human milk and pre-pregnancy body mass index (panel A) or number of feedings per 24 h (panel B). Different scales are used in the graphs, Figure S2. Plots presenting correlation between total protein concentration in human milk and pre-pregnancy body mass index (panel A), current body mass index (panel B), lean body mass (panel C) or total water content (panel D). Different scales are used in the graphs, Figure S3. Plots presenting correlation between true protein concentration in human milk and animal protein delivery (panel A), pre-pregnancy body mass index (panel B), current body mass index (panel C), lean body mass (panel D) or total water content (panel E). Different scales are used in the graphs, Figure S4. Plots presenting correlation between fat concentration in human milk and pre-pregnancy body mass index (panel A), current body mass index (panel B), lean body mass (panel C) or total water content (panel D). Different scales are used in the graphs, Figure S5. Plots presenting correlation between energy content of human milk and pre-pregnancy body mass index (panel A), current body mass index (panel B), lean body mass (panel C) or total water content (panel D). Different scales are used in the graphs.

## References

[1] Eidelman A.I., Schanler R.J., Johnston M., Landers S., Noble L., Szucs K., Viehmann L. (2012). Breastfeeding and the use of human milk. Paediatrics.

[2] Kramer M.S., Kakuma R. (2012). Optimal duration of exclusive breastfeeding. Cochrane Database Syst. Rev..

[3] Jarosz M. (2017). Normy Żywienia dla Populacji Polski.

[4] Agostoni C., Braegger C., Decsi T., Kolacek S., Koletzko B., Michaelsen K.F., Mihatsch W., Moreno L.A., Puntis J., Shamir R. (2009). Breast-feeding: A commentary by the ESPGHAN Comitee on Nutrition. J. Ped. Gastr. Nutr..

[5] Victora C.G., Bahl R., Barros A.J., Franca G.V., Horton S., Krasevec J., Murch S., Sankar M.J., Walker N., Rollins N.C. (2016). Breastfeeding in the 21st century: Epidemiology, mechanisms, and lifelong effect. Lancet.

[6] Horta B.L., de Mola C.L., Victora C.G. (2015). Long-term consequences of breastfeeding on cholesterol, obesity, systolic blood pressure and type 2 diabetes: A systematic review and meta-analysis. Acta Paediatr..

[7] WHO. https://apps.who.int/iris/bitstream/handle/10665/95585/9789241506120_eng.pdf?sequence=1.

[8] Jiang J., Wu K., Yu Z., Ren Y., Zhao Y., Jiang Y., Xu X., Li W., Jin Y., Yuan J. (2016). Changes in fatty acid composition of human milk over lactation stages and relationship with dietary intake in Chinese women. Food Funct..

[9] Ballard O., Morrow A.L. (2013). Human milk composition: Nutrients and bioactive factors. Pediatr. Clin. N. Am..

[10] Mizuno K., Nishida Y., Taki M., Murase M., Mukai Y., Itabashi K., Debari K., Iiyama A. (2009). Is increased fat content of hindmilk due to the size or the number of milk fat globules?. Int. Breastfeed. J..

[11] Pundir S., Wall C.R., Mitchell C.J., Thorstensen E.B., Lai C.T., Geddes D.T., Cameron-Smith D., ESPGHAN Committee on Nutrition (2017). Variation of human milk glucocorticoids over 24 h period. J. Mammary Gland Biol. Neoplasia..

[12] Bzikowska-Jura A., Czerwonogrodzka-Senczyna A., Olędzka G., Szostak-Węgierek D., Weker H., Wesołowska A. (2018). Maternal nutrition and body composition during breastfeeding: Association with human milk composition. Nutrients.

[13] Martin M.A., Lassek W.D., Gaulin S.J.C., Evans R.W., Woo J.G., Geraghty S.R., Davidson B.S., Morrow A.L., Kaplan H.S., Gurven M.D. (2012). Fatty acid composition in the mature milk of Bolivian forager-horticulturalists: Controlled comparisons with a US sample. Matern. Child Nutr..

[14] Bzikowska-Jura A., Czerwonogrodzka-Senczyna A., Jasińska-Melon E., Mojska H., Olędzka G., Wesołowska A., Szostak-Węgierek D. (2019). The concentration of Omega-3 fatty acids in human Milki s related to their habitual but not current intake. Nutrients.

[15] Mäkelä J., Linderborg K., Niinikoski H., Yang B., Lagström H. (2013). Breast milk fatty acid composition differs between overweight and normal weight women: The STEPS Study. Eur. J. Nutr..

[16] Leghi G.E., Netting M.J., Middleton P.F., Wlodek M.E., Geddes D.T., Muhlhausler B.S. (2020). The impact of maternal obesity on human milk macronutrient coposition: A systemic review and meta-analysis. Nutrients.

[17] Galante L., Milan A.M., Reynolds C.M., Cameron-Smith D., Vickers M.H., Pundir S. (2018). Sex-specific human milk composition: The role of infant sex in determining early life nutrition. Nutrients.

[18] Pardo I.M., Geloneze B., Tambascia M.A., Pereira J.L., Filho A.A.B. (2004). Leptin as a marker of sexual dimorphism in newborn infants. J. Pediatr..

[19] Geary M.P.P., Pringle P.J., Rodeck C.H., Kingdom J.C.P., Hindmarsh P.C. (2003). Sexual dimorphism in the growth hormone and insulin-like growth factor axis at birth. J. Clin. Endocrinol. Metab..

[20] Hinde K., Carpenter A.J., Clay J.S., Bradford B.J. (2014). Holsteins favor Heifers, not bulls: Biased milk production programmed during pregnancy as a function of fetal sex. PLoS ONE.

[21] Landete-Castillejos T., García A., López-Serrano F.R., Gallego L. (2005). Maternal quality and differences in milk production and composition for male and female Iberian red deer calves (*Cervus elaphus hispanicus*). Behav. Ecol. Sociobiol..

[22] Quesnel L., MacKay A., Forsyth D.M., Nicholas K.R., Festa-Bianchet M. (2017). Size, season and offspring sex affect milk composition and juvenile survival in wild kangaroos. J. Zool..

[23] Robert K.A., Braun S. (2012). Milk composition during lactation suggests a mechanism for male biased allocation of maternal resources in the tammar wallaby (*Macropus eugenii*). PLoS ONE.

[24] Kanazawa S., Segal N.L. (2017). Same-sex twins are taller and heavier than opposite-sex twins (but only if breastfed): Possible evidence for sex bias in human breast milk. J. Exp. Child Psychol..

[25] National Longitudinal Study of Adolescent Health. https://www.cpc.unc.edu/projects/addhealth.

[26] Lee S.G., Chung T.H. (1985). Lipid content of breast milk in Korean women. J. Korean Pediatr. Soc..

[27] Nommsen L.A., Lovelady C.A., Heinig M.J., Lönnerdal B., Dewey K.G. (1991). Determinants of energy, protein, lipid, and lactose concentrations in human milk during the first 12 mo of lactation: The DARLING Study. Am. J. Clin. Nutr..

[28] Szponar L., Wolnicka K., Rychlik E. (2011). Album of Photographs of Food Products and Dishes.

[29] Euro Who. http://www.euro.who.int/en/health-topics/disease-prevention/nutrition/a-healthy-lifestyle/body-mass-index-bmi.

[30] Groh-Wargo S., Valentic J., Khaira S., Super D.M., Collin M. (2016). Human milk analysis using mid-infrared spectroscopy. Nutr. Clin. Pract..

[31] Smilowitz J.T., Gho D.S., Mirmiran M., German J.B., Underwood M.A. (2014). Rapid measurement of human milk macronutrients in the neonatal intensive care unit: Accuracy and precision of fourier transform mid-infrared spectroscopy. J. Hum. Lact..

[32] Khan S., Hepworth A.R., Prime D.K., Lai C.T., Trengove N.J., Hartmann P.E. (2013). Variation in fat, lactose, and protein composition in breast milk over 24 h: Associations with infant feeding patterns. J. Hum. Lact..

[33] Fujimori M., França E.L., Fiorin V., Morais T.C., Honorio-França A.C., de Abreu L.C. (2015). Changes in the biochemical and immunological components of serum and colostrum of overweight and obese mothers. BMC Pregnancy Childbirth.

[34] Bravi F., Wiens F., Decarli A., Dal Pont A., Agostoni C., Ferraroni M. (2016). Impact of maternal nutrition on breast-milk composition: A systematic review. Am. J. Clin. Nutr..

[35] Chang N., Jung J.A., Kim H., Jo A., Kang S., Lee S.W., Yi H., Kim J., Yim J.G., Jung B.M. (2015). Macronutrient composition of human milk from Korean mothers of full-term infants born at 37–42 gestational weeks. Nutr. Res. Pract..

[36] Yang T., Zhang Y., Ning Y., You L., Ma D., Zheng Y., Yang X., Li W., Wang J., Wang P. (2014). Breast milk macronutrient composition and the associated factors in urban Chinese mothers. Chin. Med. J..

[37] Hahn W.-H., Song J.-H., Song S., Kang N. (2017). Do gender and birth height of infant affect calorie of human milk? An association study between human milk macronutrient and various birth factors. J. Matern. Fetal Neonatal Med..

[38] Fujita M., Roth E., Lo Y.-J., Hurst C., Vollner J., Kendell A. (2012). In poor families, mothers’ milk is richer for daughters than sons: A test of Trivers–Willard hypothesis in agropastoral settlements in Northern Kenya. Am. J. Phys. Anthropol..

[39] Fumeaux C.J.F., Garcia-Rodenas C.L., De Castro C.A., Courtet-Compondu M.-C., Thakkar S.K., Beauport L., Tolsa J.-F., Affolter M. (2019). Longitudinal analysis of macronutrient composition in preterm and term human milk: A prospective cohort study. Nutrients.

[40] Thakkar S.K., Giuffrida F., Cristina C.H., De Castro C.A., Mukherjee R., Tran L.A., Steenhout P., Lee L.Y., Destaillats F. (2013). Dynamics of human milk nutrient composition of women from Singapore with a special focus on lipids. Am. J. Hum. Biol..

[41] Xie G., Ma X., Zhao A., Wang C., Zhang Y., Nieman D., Nicholson J.K., Jia W., Bao Y., Jia W. (2014). The metabolite profiles of the obese population are gender-dependent. J. Proteome Res..

[42] De Luca A., Hankard R., Alexandre-Gouabau M.-C., Ferchaud-Roucher V., Darmaun D., Boquien C.-Y. (2016). Higher concentrations of branched-chain amino acids in breast milk of obese mothers. Nutrition.

[43] Larnkjær A., Bruun S., Pedersen D., Zachariassen G., Barkholt V., Agostoni C., Christian M., Husby S., Michaelsen K.F. (2016). Free amino acids in human milk and associations with maternal anthropometry and infant growth. J. Pediatr. Gastroenterol. Nutr..

[44] Dritsakou K., Liosis G., Valsami G., Polychronopoulos E., Skouroliakou M. (2017). The impact of maternal-and neonatal-associated factors on human milk’s macronutrients and energy. J. Matern. Fetal Neonatal Med..

[45] Prentice P., Ong K.K., Schoemaker M.H., van Tol E.A., Vervoort J., Hughes I.A., Acerini C.L., Dunger D.B. (2016). Breast milk nutrient content and infancy growth. Acta Paediatr..

[46] Gridneva Z., Tie W., Rea A., Lai C., Ward L., Murray K., Hartmann P., Geddes D. (2018). Human milk casein and whey protein and infant body composition over the first 12 months of lactation. Nutrients.

[47] Grunewald M., Hellmuth C., Kirchberg F.F., Mearin M.L., Auricchio R., Castillejo G., Korponay-Szabo I.R., Polanco I., Roca M., Vriezinga S.L. (2019). Variation and interdependencies of human milk macronutrients, fatty acids, adiponectin, insulin, and IGF-II in the European PreventCD cohort. Nutrients.

[48] Quinn E.A. (2013). No evidence for sex biases in milk macronutrients, energy, or breastfeeding frequency in a sample of Filipino mothers. Am. J. Phys. Anthropol..

[49] Casadio Y.S., Williams T.M., Lai C.T., Olsson S.E., Hepworth A.R., Ed G.D., Hartmann P.E. (2010). Evaluation of a mid-infrared analyzer for the determination of the macronutrient composition of human milk. J. Hum. Lact..

[50] Dewey K.G., Finley D.A., Lonerdal B. (1984). breast milk volume and composition during late lactation (7–20 Months). J. Ped. Gastr. Nutr..

[51] Neville M.C., Neifert M.R., Neville M.C., Neifert M.R. (1983). Lactation: Physiology, Nutrition and Breast-Feeding.

[52] Michaelsen K.F., Larsen P.S., Thomsen B.L., Samuelson G. (1994). The copen-hagen cohort study on infant nutrition and growth: Breast milk intake, human milk macronutrient content, and influencing factors. Am. J. Clin. Nutr..

[53] Powe C.E., Knott C.D., Conklin-Brittain N. (2010). Infant sex predicts breast milk energy content. Am. J. Hum. Biol..

[54] Tamimi R.M., Lagiou P., Mucci L.A., Hsieh C.C., Adami H.O., Trichopoulous D. (2003). Average energy intake among pregnant women carrying a boy compared with a girl. Br. Med. J..

[55] Heyward V.H., Stolarczyk L.M. (1996). Applied Body Composition Assessment.

[56] Rakicioglu N., Samur G., Topcu A., Topcu A.A. (2006). The effect of ramadan on maternal nutrition and composition of breast milk. Pediatr. Int..

[57] Innis S.M. (2014). Impact of maternal diet on human milk composition and neurological development of infants. Am. J. Clin. Nutr..

