# Supplementary material for: Impact of Infant and Maternal Factors on Energy and Macronutrient Composition of Human Milk
Source: Nutrients. 2020 Aug 26;12(9):2591. doi: 10.3390/nu12092591 (PMC7551856; doi:10.3390/nu12092591)
Supplement: Supplementary file 1 [file nutrients-12-02591-s001.pdf]

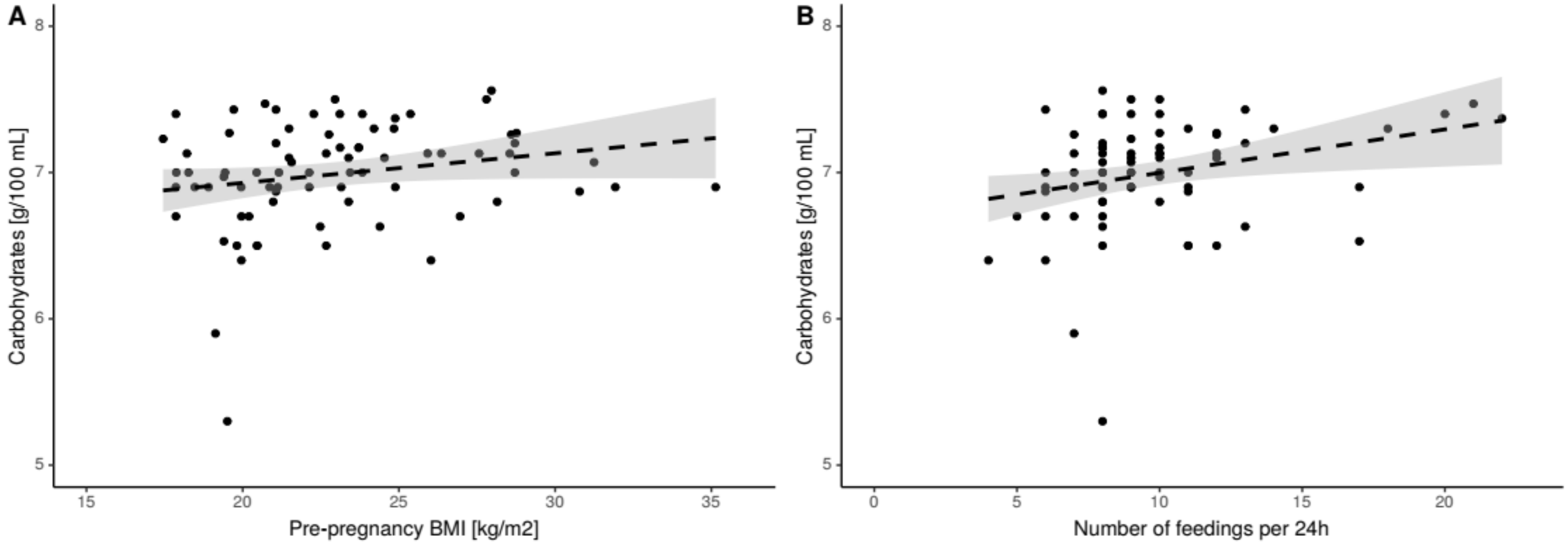

Figure S1. Plots presenting correlation between carbohydrates concentration in human milk and pre-pregnancy body mass index (panel A) or number of feedings per 24 hours (panel B). Different scales are used in the graphs.

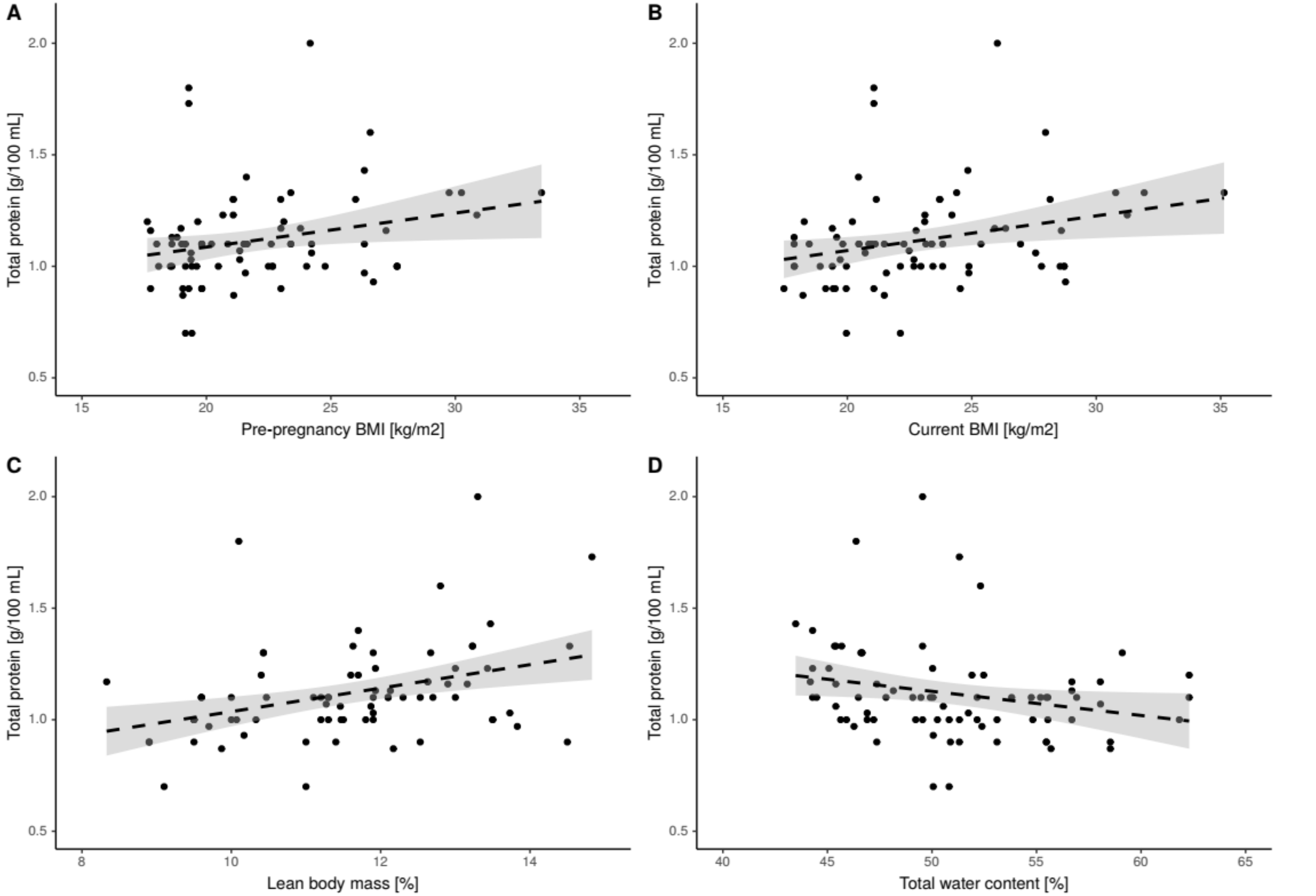

Figure S2. Plots presenting correlation between total protein concentration in human milk and pre-pregnancy body mass index (panel A), current body mass index (panel B), lean body mass (panel C) or total water content (panel D). Different scales are used in the graphs.

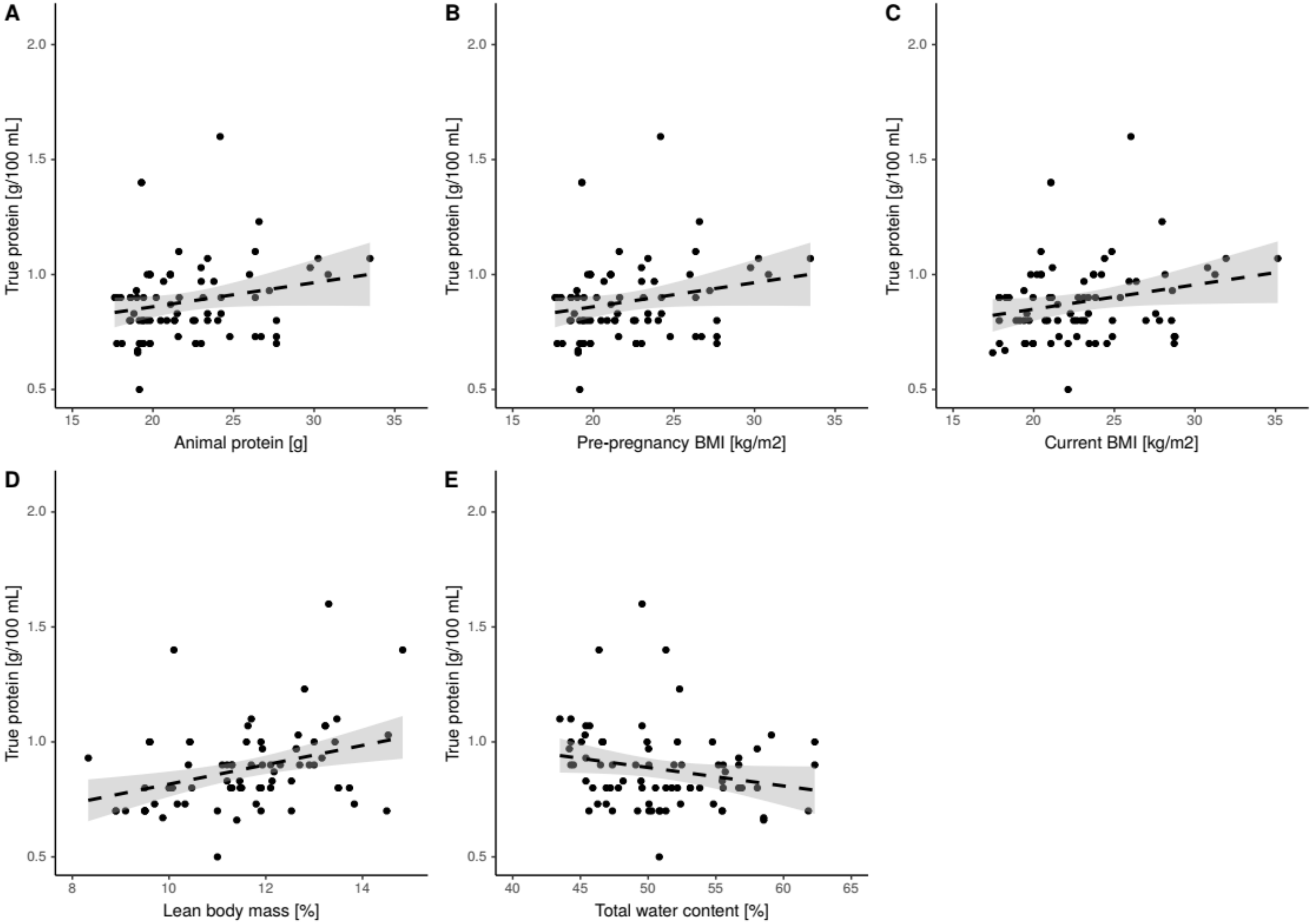

Figure S3. Plots presenting correlation between true protein concentration in human milk and animal protein delivery (panel A), pre-pregnancy body mass index (panel B), current body mass index (panel C), lean body mass (panel D) or total water content (panel E). Different scales are used in the graphs.

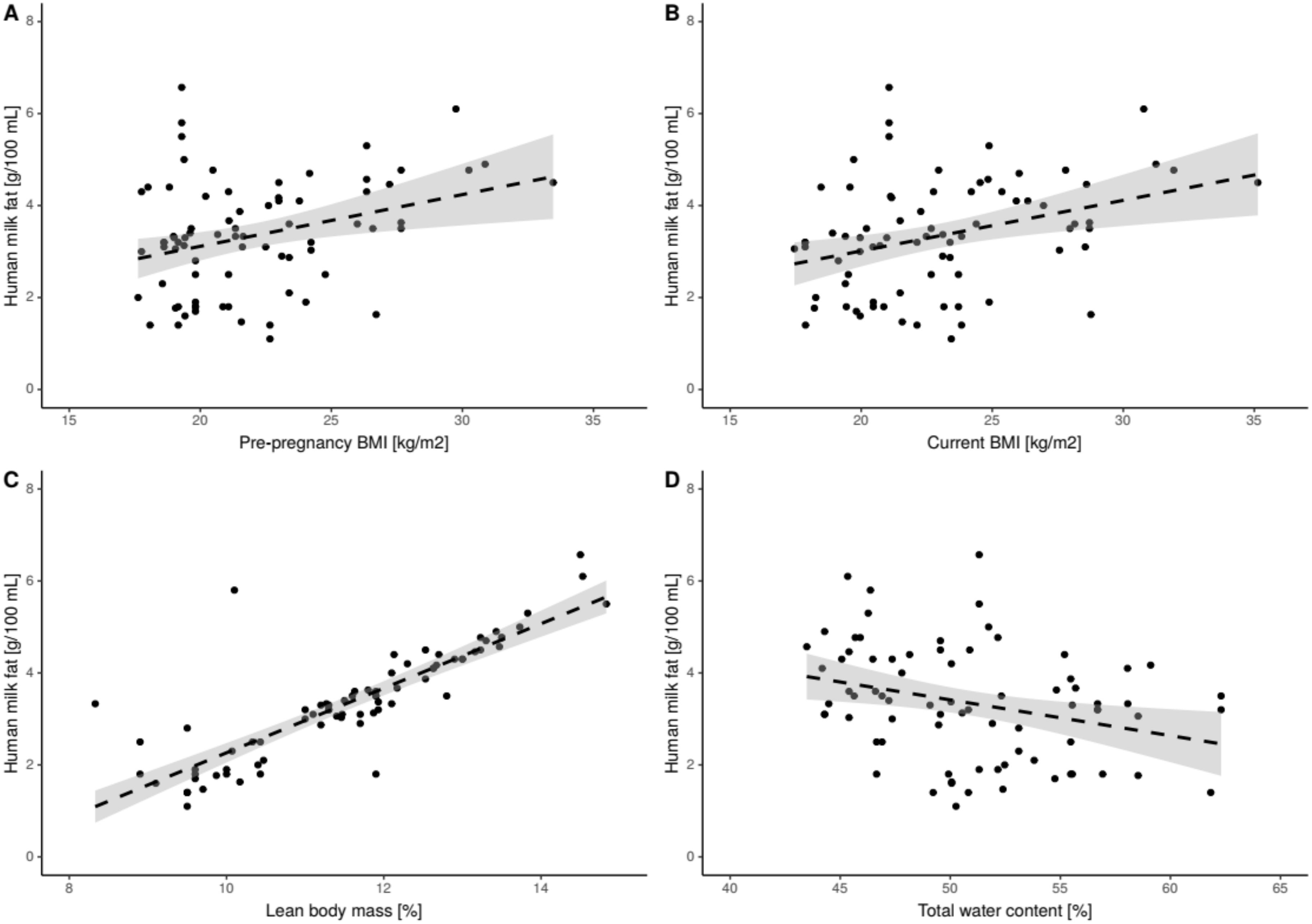

Figure S4. Plots presenting correlation between fat concentration in human milk and pre-pregnancy body mass index (panel A), current body mass index (panel B), lean body mass (panel C) or total water content (panel D). Different scales are used in the graphs.

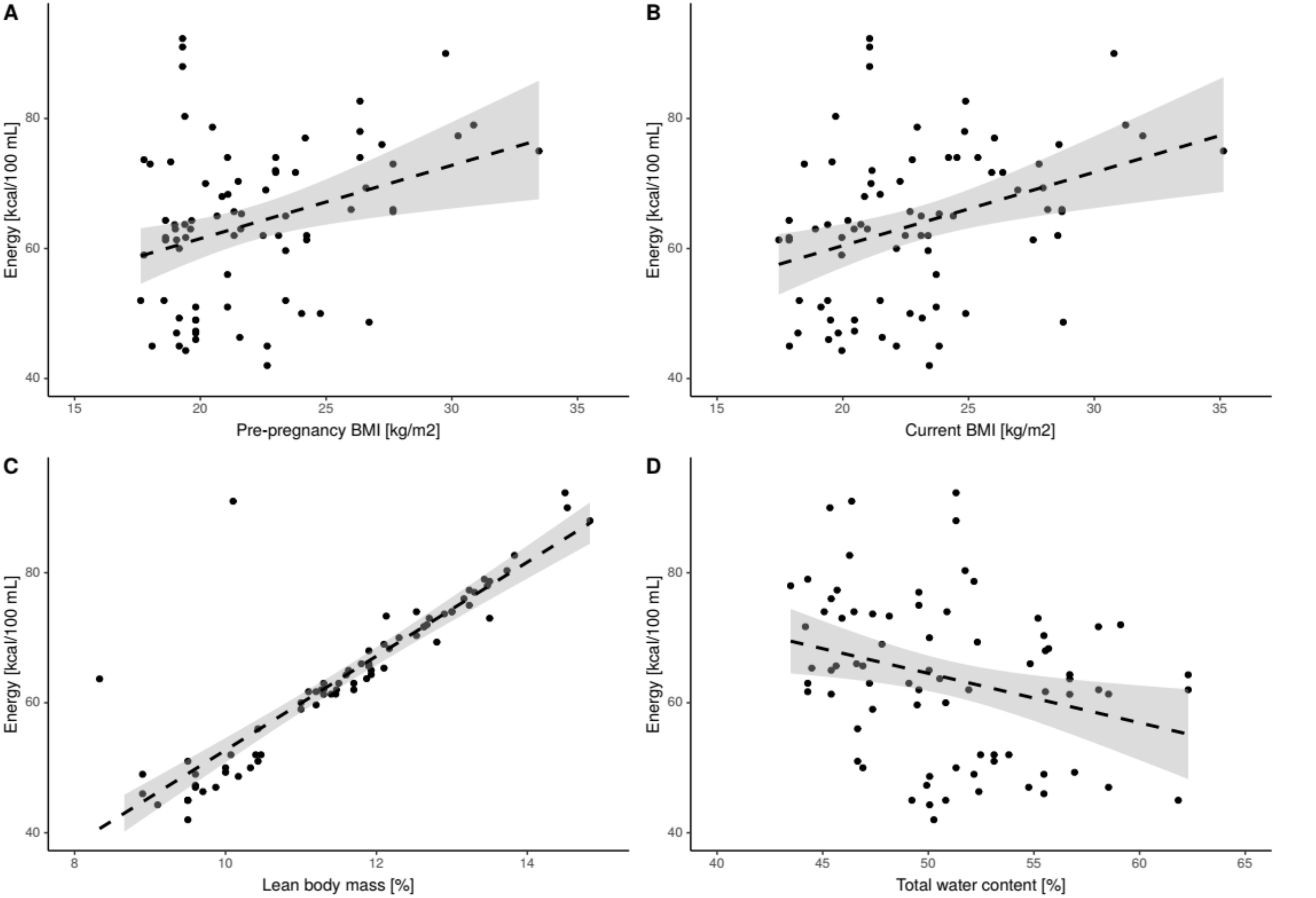

Figure S5. Plots presenting correlation between energy content of human milk and pre-pregnancy body mass index (panel A), current body mass index (panel B), lean body mass (panel C) or total water content (panel D). Different scales are used in the graphs.
